# Knowledge and use of emergency contraception among students of public secondary schools in Ilorin, Nigeria

**DOI:** 10.11604/pamj.2016.23.74.8688

**Published:** 2016-03-10

**Authors:** Oluwole Adeyemi Babatunde, Demilade Olusola Ibirongbe, Owen Omede, Olubukola Oluwakemi Babatunde, Kabir Adekunle Durowade, Adekunle Ganiyu Salaudeen, Tanimola Makanjuola Akande

**Affiliations:** 1 Department of Community Medicine, Federal Medical Center, Ido-Ekiti, Nigeria; 2 Department of Epidemiology and Community Health, University of Ilorin, Ilorin, Nigeria

**Keywords:** Abortion, adolescence, emergency contraception, unwanted pregnancy

## Abstract

**Introduction:**

Unintended pregnancy and unsafe abortion pose a major reproductive health challenge to adolescents. Emergency contraception is safe and effective in preventing unplanned pregnancy. The objective of this study was to assess the student's knowledge and use of emergency contraception.

**Methods:**

This cross-sectional study was carried out in Ilorin, Nigeria, using multi-stage sampling method. Data was collected using pre-tested semi-structured self-administered questionnaire. Knowledge was scored and analysed. SPSS version 21.0 was used for data analysis. A p-value <0.05 was considered statistically significant.

**Results:**

27.8% of the respondents had good knowledge of emergency contraception. Majority of respondents (87.2%) had never used emergency contraception. Majority of those who had ever used emergency contraception (85.7%) used it incorrectly, using it more than 72 hours after sexual intercourse (p=0.928).

**Conclusion:**

Knowledge about Emergency contraception and prevalence of use were low. Contraceptive education should be introduced early in the school curriculum for adolescents.

## Introduction

Teenage sexual activity is increasing globally with a trend towards early onset [1–3]. As many as 20-50% of adolescents have initiated sexual activity with age at first sexual intercourse ranging from fourteen to eighteen years [4, 5]. Unintended pregnancy therefore poses a major challenge to the reproductive health of these young adults [1, 3–6]. In a study in Nigeria, in 2003, as much as 5% of all sexually active adolescent girls have been pregnant all of which were unintended; leading to an induced abortion rate of one hundred percent [7]. Similarly, in the United States in the year 2000, adolescents had over eight hundred thousand pregnancies out of which 85% were unintended [8]. Among the various forms of contraception, Emergency Contraceptive pills (EC) can be used after sexual intercourse, offering a second chance to prevent unwanted pregnancy [6]. It is a safe, effective and relatively inexpensive way to prevent pregnancy, after unplanned or unprotected sexual intercourse [9–11], preventing approximately 80-85% of pregnancies that would otherwise occur [12, 13]. EC is appropriate for adolescents who engage in sporadic and occasional sexual intercourse, and especially among those who find regular contraceptives intolerable or who use them sparingly [11]. A Nigerian community-based study of abortion prevalence found that one-third of the women who obtained an abortion were adolescents [14]. Similarly, hospital-based studies have shown that in Nigeria, up to eighty percent of those with abortion-related complications are adolescents [15, 16]. The performance of abortion is illegal under Nigerian criminal law, unless the woman's life is threatened by the pregnancy. As a result, most induced abortions are usually done by “quacks”, usually obtained clandestinely, and are frequently unsafe [1, 2]. One of the major factors responsible for unwanted pregnancies and unsafe abortion is lack of knowledge of the various methods of contraceptives available, including EC. Findings among nurses and nursing students in Kenya showed that only 48% of respondents have heard of EC [17]. Reports from Nigeria and the United States also showed generally low level of awareness of EC among adolescents [18–20]. Various misconceptions about EC among teenagers were reported in a related study in England; these ranged from seeing it as abortifacient to seeing it as a poison [3]. Previous studies in Africa and Europe reported varying levels of awareness about EC ranging from 48-93% [6, 11, 17, 18, 21–24]. Among undergraduates in Lagos, Nigeria, Ebuehi, et al., reported that 36% of respondents correctly identified emergency contraceptives [18]. Different levels of usage of EC have been reported among sexually active adolescents. Percentage use among respondents of 2-30% has been reported in previous studies. [6, 11, 17, 21, 22]. When asked if they were willing to use EC in the future, 23% of those familiar with EC intend to use it in the future in Kenya [17]. A similar trend was reported in Nigeria where approximately 37% of respondents would like to use EC in the future, 58% would not, while 5% were unsure [11]. Fifty-three percent of sexually active respondents were willing to recommend EC to friends [17]. Studies have shown that fear of side effects of modern contraceptives was the most common reason for non-use of EC [11]. In a similar study among women in England, 43% expressed fears of the increased health risks of EC [25]. Few studies have been published on awareness and use of EC among adolescents, especially in the north central region of Nigeria. The objective of this study was to assess the student's knowledge and use of EC in public secondary schools in Ilorin, Nigeria.

## Methods

The study was carried out in Ilorin metropolis, in the north central region of Nigeria. There are 53 mixed-sex public secondary schools within the 3 Local Government Areas (LGA) that make up Ilorin metropolis, distributed as follows: Ilorin East (22 schools), Ilorin West (17) and Ilorin South (14). Currently, the projected population of Ilorin metropolis is 854,537. Adolescents make up 18% of the total population. A multi-stage sampling method was applied to select respondents. In the first stage, one out of the three LGAs was selected using simple random method. Ilorin South LGA was selected. In the second stage, three wards were selected using simple random method from the selected LGA. The third stage involved selecting a public school each from each of the three wards. The fourth stage consisted of selection of 100 students from each of the three schools using systematic random sampling. This was done without giving consideration to academic class: junior or senior class, science, art or commercial class. Students aged 10 to 19 years at the time of the study and who were enrolled for at least an academic session, which runs for a year, were included in the study. A sample size of 273 was determined using Fischer's formula [26] with an alpha of 0.05. The study was a cross-sectional study using a pre-tested semi-structured, self-administered questionnaire. The questionnaire had three parts: the first section elicited baseline information on the socio-demographic characteristics of respondents, the second section elicited information on knowledge of EC including indication, time frame for use, and side effects like nausea, vomiting, and delayed menstruation. The final section assessed use of EC among respondents. Dependent variables included proportion of respondents with correct knowledge about EC, proportion that are sexually active, and proportion of respondents who have used EC. The reliability of the instrument was guaranteed by a test-retest method. The correlation coefficient was 0.8. In addition to the foregoing, methodological triangulation was done with two separate research assistants using the same questionnaire to interview the same respondent. The findings were then compared. The content validity of the questionnaire was determined by a panel of consultants and health education expert from the Department of Epidemiology and Community Health, University of Ilorin Teaching Hospital, Ilorin.

Statistical Package for Social Sciences (SPSS) software version 21.0 was used to generate frequency tables and percentages. Cross-tabulation of variables was also done. Chi-squared test was used to test for significant associations between variables that were nominal. Yates’ chi square was used when an expected cell contained less than 5 respondents in 20% of the cells. Student's t-test and ANOVA were used to test between means. A p-value of less than 0.05 was considered as statistically significant. During analysis, knowledge was scored. Sections of the questionnaire testing knowledge of susceptibility to sexual activity and pregnancy, knowledge of severity of pregnancy as an adolescent, knowledge of benefit of use of emergency contraceptive, and knowledge of barrier to the use of EC added up the score of knowledge. In addition to these, knowledge about side effects, correct time frame, and indication for use of EC and other important aspects were all added together. Correct knowledge in each variable was scored one while incorrect knowledge was scored zero. There were a total of 55 questions. The maximum score was 55 while the minimum score was 0. All the respondents who were not aware of EC were given a score of zero. The range of scores (0-55) was further divided into three equal groups for ease of analysis. Those that fell within 0-17 were termed to have poor knowledge, those scoring 18-35 were termed to have fair knowledge, and those scoring 36-55 were termed to have good knowledge. Ethical approval for the study was obtained from the ethical committee of University of Ilorin Teaching Hospital, Ilorin as well as the Kwara State Schools Management Board and Ministry of Education. Written Informed consent was sought from the respondents aged 18 and above. Written informed consent from parents and/or guardian was obtained for students less than the age of 18 years at the time of the study. The consent of the parent-teacher association was also sought as part of the advocacy process. The strength of this study is that it provides information on usage of emergency contraceptives among adolescents which would be helpful in policy-formulation and planning for adolescents’ reproductive health services. A limitation of the study is that knowledge results were not analyzed by age or gender which would have given more information on the characteristics of adolescents with good knowledge of EC.

## Results

**Figure 1:**
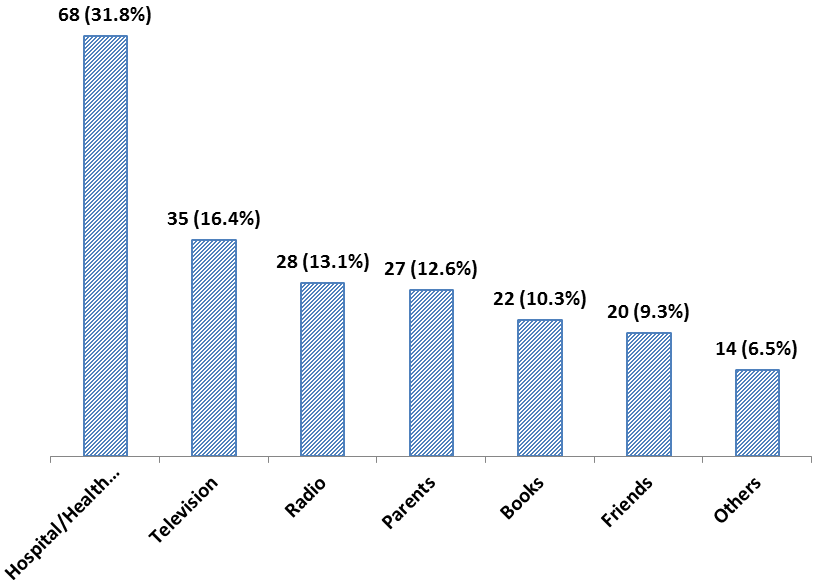
Source of first knowledge about emergency contraception

Table 1 shows the basic socio-demographic characteristics of the respondents. Early adolescents aged 10-14 years age group accounted for about half of the sample. The proportion of female respondents was slightly higher. There were more Moslem respondents than Christian as seen in Table 1. About half of all respondents had fair knowledge based on scores (Table 2). The major source of first knowledge about EC was health workers and hospitals. This was followed by the mass media (Television and Radio) as shown in Figure 1. Those that had correct knowledge of the indication of EC are 75.8% while those that had correct knowledge of time frame of EC are 66.7%. The majority of the respondents had correct knowledge of what EC is and its side effects. The majority of respondents (87.2%) had never used EC, and majority of those who had ever used it (85.7%) used it incorrectly, using it more than 72 hours after sexual intercourse (p=0.928), while 5 (14.3%) used it correctly, using it within 72 hours of coitus. For those who had sexual intercourse once in the last three months, 30.4% had ever used EC, while for those who had sexual intercourse twice or more in the last three months, 48.3% had ever used EC. More respondents who had good knowledge of EC had ever used it compared to respondents who had poor knowledge of EC. The difference was not statistically significant (Table 3).

**Table 1:** Age, sex and religion of respondents

	Frequency (%)
	N=273
**Socio-demographics**	
**Age (years)**	
10-14 (Early)	138 (50.5)
15-19 (Late)	135 (49.5)
**Mean Age (years)**	14.52 ± 1.86
**Sex**	
Male	136 (49.8)
Female	137 (50.2)
**Religion**	
Christian	127 (46.5)
Islam	146 (53.5)

**Table 2:** Knowledge about emergency contraception

Knowledge score	Frequency (%)
Poor (0-17)	59 (21.6)
Fair (18-35)	143 (52.4)
Good(36-55)	71 (26.0)
**Total**	**273 (100.0)**
Mean Knowledge Score (SD)	26.7±14.4
**Indication, time-frame**	**Frequency (%)**
**& side-effects of EC**	**N=273**
**Indication**	
Incorrect	66 (24.2)
Correct	207 (75.8)
**Time Frame**	
Incorrect	91 (33.3)
Correct	182 (66.7)
**Side Effect**	
Incorrect	75 (27.5)
Correct	198 (72.5)
**What is EC**	
Incorrect	71 (26.0)
Correct	202 (74.0)

**Table 3:** Sexual activity and use of emergency contraception

	Ever used EC		
	Yes	No	
**Sexual activity**			
**Times engaged in sex in the past 3 months**			
0 - 1	7 (30.4)	16 (69.6)	**p =0.162**
≥ 2	28 (48.3)	31 (51.7)	
Total	**35**	**47**	*82
**Sexual partners since sexual initiation**			
1 – 2	32 (47.1)	36 (52.9)	**p =0.024**
≥ 3	3 (23.1)	11 (76.9)	
Total	**35**	**47**	*82
**Knowledge score**			
**Poor (0-17)**	6 (10.2)	53 (89.8)	
**Fair (18-35)**	18 (12.6)	125 (87.4)	**p =0.420**
**Good (36-55)**	11 (15.5)	60 (84.5)	
Total	**35**	**238**	**273

* The total number of respondents who ever had sex=82, out of which 35 ever used EC

** The total number of respondents=273, out of which 35 ever used EC

## Discussion

In our sample, 12.8% of adolescents aged 10-19 years had ever used EC. This is comparable to findings from studies in Benin, Nigeria and Nairobi, Kenya [6, 17] which also reported low prevalence. This study found poor knowledge of EC amongst the adolescents. Only 27.8% of the respondents had good knowledge of EC, though this was statistically significantly related to its usage. This may be due to the traditional societal poor attitude to adolescent sexuality in Nigeria. Higher prevalence was reported in studies done in more developed countries [21, 22]. Developed countries are known to have better information systems and health awareness when compared to developing nations. Awareness education campaigns should be encouraged and instituted to improve knowledge and use of EC among adolescents, especially in developing countries. In this study, more of the respondents (31.8%) learnt about EC through hospital or health workers while 16.4% heard about EC for the first time from television. This limited source of information might be the cause of poor awareness among the students. Therefore, all stakeholders such as parents, teachers and the government should take an active role in properly educating adolescents on sex at an early age, and through the use of various media appropriate to them. This study suggests that benefitting from EC was generally low among respondents, as shown by the low prevalence of EC use. Also another study among young women who had previously had clandestine abortions in Nigeria showed that only 16% of them had used EC [14]. These studies suggest that the majority of adolescents in Nigeria have low knowledge of the benefits of using EC. There is therefore the need to scale-up teachings on the benefits of using EC locally especially in the developing nations that have observed low use. Among the respondents that were aware about EC, knowledge of the correct time-frame within which it should be used was generally poor (33.3%). This was similar to findings in previous studies done in south west Nigeria, Benin-city, and Lagos [6, 11, 18]. Poor knowledge of correct time-frame of EC use might inhibit someone who could still prevent a pregnancy from taking EC because they thought they had missed their “window” of effectiveness [6]. A study on EC label comprehension for teens found that 79% or more of adolescents aged 12-17 correctly understood six key concepts found in labeling (EC prevents pregnancy after unprotected sex, it should be taken as soon as possible, it should be taken within 72 hours, it should not replace regular contraception, it does not protect against sexually transmitted infections (STIs), it should not be used by women who are already pregnant) [27]. Cremer et al. found that older teens (aged 16-17) were a bit more likely to understand the key comprehension points of the study than the younger girls (aged 12-15), but the younger girls understood a majority of the points with very high accuracy, including the reason to use EC and that it does not protect against HIV/AIDS [28]. Our study however did not consider age difference in knowledge. Few of our respondents (27.5%) had knowledge of side-effects of EC. This figure is lower compared to that reported in a study in England [25]. A small proportion of respondents (12.8%) had ever used emergency contraception. Low prevalence was generally reported in population-based surveys among youths aged 15-24 years, from developing countries [29]. The higher prevalence in developed countries might be due to the level of awareness among adolescents about EC, and the cultural norms and reproductive health policies in different countries. For example in the United Kingdom and Canada, young people can purchase EC without a prescription. This is also true for France, where ado¬lescents can access EC from a pharmacy free of charge without parental approval or a doctor's prescription. However, the pharmacist is required to educate adolescents about EC and encour-age them to visit a doctor [30]. In Nigeria, the societal attitude to adolescent sexuality may be responsible for the poor access of the adolescents to EC.

## Conclusion

It can be concluded from this study that knowledge about EC was fair while students’ past usage of EC was low. Health education intervention about knowledge and use of EC is therefore important in adolescent sexual education. There is need to incorporate adolescent-friendly services into Primary Health Care system with easy access to EC. At the level of the National Government, there is need for introduction of sexual education classes at an earlier stage. Also there is need for a policy to incorporate sexuality education in the secondary school curriculum including education on EC. This will help to reduce misconceptions about EC and improve health benefit from use of EC.

### What is known about this topic

- Teenage sexual activity and unintended pregnancy is on the rise.
- Emergency contraception is safe and effective to prevent unintended pregnancy.
- Emergency contraception is appropriate for adolescents.

### What this study adds

- Knowledge of emergency contraception is low.
- Prevalence of emergency contraception use is low.
- Emergency contraceptives are incorrectly used by many adolescents.
